# Association of Urea-to-Creatinine Ratio with Functional Outcomes in Patients with Traumatic Brain Injury

**DOI:** 10.3390/jcm15124766

**Published:** 2026-06-19

**Authors:** Valentina Blažinčić, Anđela Grgić, Kristina Kralik, Ivica Ščurić, Ivana Klepo, Duško Cerovec

**Affiliations:** 1 Department of Craniocerebral Rehabilitation and Acute Neurology, Special Hospital for Medical Rehabilitation Krapinske Toplice, 49217 Krapinske Toplice, Croatia; ivica.scuric@sbkt.hr (I.Š.);; 2 Faculty of Medicine, Josip Juraj Strossmayer University of Osijek, 31000 Osijek, Croatia; 3 Department of Physical Medicine and Rehabilitation, Clinical Hospital Centre Osijek, 31000 Osijek, Croatia; 4 School of Medicine, University of Zagreb, 10000 Zagreb, Croatia; 5 Department of Occupational Therapy, University of Applied Health Sciences, 10000 Zagreb, Croatia; 6 Department of Social Work, Faculty of Law, University of Zagreb, 10000 Zagreb, Croatia; 7 Department of Cardiologic Rehabilitation, Special Hospital for Medical Rehabilitation Krapinske Toplice, 49217 Krapinske Toplice, Croatia

**Keywords:** traumatic brain injury, functional independence measure, urea-to-creatinine ratio

## Abstract

**Highlights:**

**What are the main findings?**

- Patients with UCRs ≥ 80 had a significantly lower discharge FIM compared to patients with UCRs < 80.
- The significant independent predictors of discharge FIM were: the nutritional route (NGT/PEG), level of consciousness, and FIM at admission. The UCR did not remain independently associated with the discharge FIM.

**What are the implications of the main findings?**

- During inpatient rehabilitation, monitoring the UCR is suggested as a marker of global catabolic burden with a possible association with the outcome.
- In TBI patients, the nutritional route (NGT/PEG), level of consciousness, and FIM at admission provide additional prognostic information regarding patient functional outcomes, which is valuable for guiding patients’ families.

**Abstract:**

**Background**: In patients with traumatic brain injury (TBI), proteins are considered the main source of energy. Previous studies have suggested that an increase in the urea-to-creatinine ratio (UCR) indicates the onset of protein catabolism. Therefore, we aimed to investigate the associations of the UCR with the functional independence measure (FIM). **Methods**: This single-center retrospective study included 291 patients aged 17–87 years who underwent inpatient rehabilitation within the first 6 months post-TBI. Their demographic, clinical, neuroradiological, and laboratory data (eGFR, urea, creatinine, UCR) were collected. Spearman’s correlation and hierarchical multivariate regression analyses adjusted for clinical covariates were performed. **Results**: The strongest significant positive correlation was found between the Glasgow Coma Scale (GCS) and FIM at admission (ρ = 0.488, *p* < 0.001) and between GCS and FIM at discharge (ρ = 0.340, *p* < 0.001). A significant negative correlation was found between the discharge UCR and FIM at discharge (ρ = −0.262, *p* < 0.003), as well as with the change in FIM (ρ = −0.207, *p* < 0.02). Patients with UCRs ≥ 80 had a significantly lower discharge FIM compared to patients with UCRs < 80 (median 27 vs. 40; *p* = 0.02). The significant independent predictors of discharge FIM were the nutritional route (NGT/PEG), level of consciousness, and FIM at admission. The UCR did not remain independently associated with the discharge FIM (ΔR^2^ = 0.004, Cohen’s f^2^ = 0.014). **Conclusions**: Although UCR is associated with functional outcomes measured by FIM in TBI patients, it is not an independent predictor of these outcomes but rather a biomarker of catabolic burden.

## 1. Introduction

Traumatic brain injury (TBI), an acute condition that can progress to a chronic disease [1,2] is associated with unresolved challenges in prevention, acute treatment, rehabilitation, and the management of long-term consequences [1]. Although the primary deficit in TBI is global neurological dysfunction, and patients present with major neurological disorders, including those of consciousness [3], primary neurological injury and its secondary sequelae significantly affect peripheral organs, including the respiratory, cardiovascular, and gastrointestinal systems [4,5,6,7]. The overall combined rate of extracranial complications can reach up to 82% in patients with TBI [7]. In the course of inpatient rehabilitation, complications persist and require optimal management [8] as their treatment directly impacts patient outcome [9,10]. One of the most prevalent complications seen in the TBI population is malnutrition, which is also closely associated with outcomes [11]. Up to 68% of patients with severe TBI develop signs of malnourishment and loss of 10–29% of their weight [12]. Complex endocrine changes influenced by the structural and functional alterations following brain trauma [13] cause hypermetabolism and catabolism, which subsequently leads to increased energy expenditure and loss of lean body mass [14]. Decreased protein synthesis and increased protein breakdown, along with concurrent changes in clinical and functional status, result in highly dynamic shifts in the nutritional status of these patients [14]. Notably, in the first month after injury, the mean energy expenditure, expressed as a percentage of the predicted value, ranges from 75% to 200% in TBI patients compared to patients without TBI [15]. In the second month after injury, energy intake increases by 21% in patients with severe TBI [12], coinciding with the period when patients are most often transferred to rehabilitation wards. Patients with TBI experience energy and protein deficits during intensive care and after discharge [16], with skeletal muscles serving as the primary protein reservoirs [17]. Recently, authors have suggested that an increase in serum urea values and a decrease in serum creatinine values- meaning an increase in their ratio -serves as an indicator for the onset of catabolism in critically ill patients [18], patients after major trauma [19], and also in non-critically ill patients [20]. In light of this, we aim to investigate the potential association of the urea-to-creatinine ratio as a biomarker of functional outcome during the first 6 months following TBI.

## 2. Materials and Methods

### 2.1. Study Design and Setting

This retrospective observational study was approved by the ethics committees of the Special Hospital for Medical Rehabilitation Krapinske Toplice (identification code 05-189/2-2025, dated 6 June 2025) and Faculty of Medicine, Josip Juraj Strossmayer University of Osijek (identification code 2158-61-46-26-54). It adhered to the latest revision of the Declaration of Helsinki. The study was conducted at the Department of Craniocerebral Rehabilitation and Acute Neurology, Special Hospital for Medical Rehabilitation Krapinske Toplice, where consecutive patients aged 17 years or older admitted to the Department between June 2012 and December 2018 were screened (n = 319) for inclusion in the study. Patients were included if they underwent inpatient rehabilitation (IR) after TBI (within the first 6 months post-injury), had a documented Glasgow Coma Scale (GCS) score and available head computed tomography (CT) findings on the day of injury, routine laboratory testing of serum urea and creatinine concentrations at the Special Hospital for Medical Rehabilitation Krapinske Toplice within 3 to 5 days of admission to IR, a functional independence measure (FIM) evaluation within 5 to 7 days of admission to IR, and an FIM re-evaluation 5 to 7 days prior to IR discharge. In the first step of analysis, we excluded patients who did not have a diagnosis of TBI (anoxic brain damage, n = 22) or had co-occurring TBI and anoxic brain damage (n = 2) according to their medical documentation. No patients met the following exclusion criteria: active gastrointestinal bleeding at rehabilitation admission, prior diagnosis of chronic kidney or liver dysfunction, corticosteroid therapy in their medical documentation, and with symptoms of hypovolemia at admission (evaluated by systolic BP < 110 mmHg, dry mouth, and dry skin) (n = 0) [21]. In the second step of analysis, we excluded patients with decreased kidney function, which was defined as an estimated glomerular filtration rate (eGFR) < 90 mL/min/1.73m^2^ (n =24). Laboratory analyses comparing admission and discharge values were restricted to patients with paired available measurements at both timepoints; therefore, the sample size was 137 for creatinine and 122 for urea/UCR.

### 2.2. Data Collection and Outcome Measures

Demographic, clinical, neuroradiological, and laboratory data were obtained from the hospital’s electronic and paper medical records. The variables included demographic characteristics (age, sex); laboratory parameters (serum urea and creatinine concentrations); eGFR; UCR; and clinical information (primary diagnosis, Glasgow Coma Scale (GCS) score, neurological impairment (level of consciousness), tracheostomy status, presence of a nasogastric tube (NGT) or percutaneous gastrostomy (PEG), and FIM evaluation). Laboratory analyses of serum urea and creatinine concentrations were performed at the laboratory of the Special Hospital for Medical Rehabilitation Krapinske Toplice. Blood samples were systematically collected in the morning (7–9 a.m.) following an overnight fast. Serum creatinine and urea concentrations were measured photometrically using standardized biochemical assays (UV photometry with urease and glutamate dehydrogenase for urea, and continuous photometry with alkaline picrate for creatinine). This analysis measured urea in mmol/L and creatinine in μmol/L. For each patient, the UCR was calculated using the following formula: UCR was calculated using the following formula; UCR = serum urea in mmol/L/[serum creatinine in μmol/L/1000]. We set the UCR cut-off at 80 mmol/L based on previous research demonstrating its association with a higher risk of in-hospital mortality and other adverse effects in critically ill patients [22]. In the TBI rehabilitation setting, this threshold should be interpreted as exploratory and requires external validation. A UCR (mostly reported in European studies) of approximately 80 mmol/L is equivalent to a blood urea nitrogen (BUN)-to-creatinine ratio (mostly reported in the United States) of  ≥20 in mg/dL, since the BUN ratio value is approximately half that of blood urea [22]. eGFR was calculated using the Chronic Kidney Disease–Epidemiology Collaboration Creatinine Equation 2021 [23,24]. Regarding functional outcomes, a trained occupational therapist evaluated the FIM within the first 5 to 7 days post-admission and again prior to discharge. To define the minimal clinically important difference for the FIM instrument, we used an FIM change score of 22, based on a study that defined the minimal clinically important difference in stroke patients [25] and a TBI study that investigated functional motor improvement during inpatient rehabilitation [26]. The included patients were categorized in groups according to TBI severity assessed using the GCS scale [27] and were classified as having mild (GCS score of 13–15), moderate (GCS score of 9–12) or severe TBI (GCS score 3–8) [28]. Regarding the level of consciousness, we classified patients into a group with normal consciousness or a group with disorders of consciousness (DoC) that included coma, i.e., the complete absence of arousal and awareness [29]; vegetative state or unresponsive wakefulness syndrome, which is defined as arousal without awareness [30]; and a minimally conscious state that is defined as fluctuating awareness [31]. We also categorized patients according to their feeding route (oral feeding group vs. NGT or PEG), presence of tracheostomy, and head CT findings. The primary outcome of this study was functional outcome at discharge, assessed using the FIM with a particular focus on the influence of the UCR. Secondary variables included demographic data (age and sex), severity of traumatic brain injury, neuroradiological findings from the initial brain CT and clinical status indicators (time from injury to rehabilitation admission, renal function (eGFR), nutritional route (oral feeding vs. NGT/PEG), level of consciousness, tracheostomy status, surgical treatment, and admission FIM.

### 2.3. Statistical Analysis

Categorical variables are presented as absolute and relative frequencies, and continuous variables are presented as the median and interquartile range (IQR) due to their non-normal distribution according to a Shapiro–Wilk test. Comparisons between independent groups were performed using a Mann–Whitney U test, and within-group changes were analyzed using the Wilcoxon signed-rank test, with corresponding 95% confidence intervals (CIs) for median differences (Hodges–Lehmann estimator). Correlations between variables were assessed using Spearman’s rank correlation coefficient (ρ). Hierarchical regression analysis was performed to assess predictors of FIM at discharge. Multivariate models were adjusted for age, sex, GCS, time from injury to rehabilitation admission, renal function (eGFR), nutritional route (oral feeding vs. NGT/PEG), level of consciousness, tracheostomy status, surgical treatment, neuroradiological characteristics and admission FIM. Model assumptions and multicollinearity were evaluated using standard diagnostic procedures and variance inflation factors (VIF), respectively. Missing data were handled using a complete-case approach. Analyses were performed using available observations for each variable, and no imputation procedures were applied. All *p* values were two-sided and the significance level was set at alpha = 0.05. All analyses were performed using MedCalc^®^ Statistical Software version 23.5.2 (MedCalc Software Ltd., Ostend, Belgium; https://www.medcalc.org; 11 June 2026).

## 3. Results

### 3.1. General Demographics and Clinical, Neuroradiological and Functional Characteristics

In the first step of the analysis 291 patients with TBI met the inclusion criteria. There were 239 males (82.1%) and a median age at inpatient rehabilitation of 45 years (range: 17 to 87 years). During inpatient rehabilitation, there were three fatalities (1%). The median time from TBI to inpatient rehabilitation admission was 66 days (interquartile range: 49 to 97), and the median duration of rehabilitation was 82 days (interquartile range: 42 to 175 days). The median initial GCS score was 7 (interquartile range: 4 to 11). At the time of injury, 177 patients (63%) were classified as having severe traumatic brain injury and 45 patients (16%) as having moderate TBI. At the time of inpatient rehabilitation, the majority of patients, n = 235 (80.8%) were conscious, while 56 (19.2%) presented with DoC. The median admission FIM was 37 (interquartile range: 20 to 95). Neuroradiological findings from head CT revealed brain contusion in 219 patients (75.8%), subarachnoid hemorrhage in 199 patients (68.9%), and subdural hematoma in 162 patients (56.1%) (Table 1).

### 3.2. Changes in Laboratory Values at Admission and Discharge

During the second step of the analysis, the sample size was found to be 137 for creatinine and 122 for urea/UCR. We found significantly higher serum creatinine and urea levels at discharge compared to those at admission (Wilcoxon test, *p* = 0.001) with the difference in median serum creatinine being the greatest (Hodges–Lehmann estimate: 5), and the difference in UCR being non-significant (Table 2).

### 3.3. Changes in FIM Value During the Rehabilitation Period

For all patients, we found a significant change in the FIM from rehabilitation to discharge (median 93 vs. 37) (Wilcoxon test, *p* < 0.001). The most significant change in FIM was observed in the subgroup of patients without DoC (Table 3).

### 3.4. Impact of Surgical or Non-Surgical Treatment on UCR and Functional Outcome Measure Through FIM

The patients who received conservative treatment during acute care had significantly higher median FIM values at admission and discharge compared to patients who underwent neurosurgical operative treatment (Mann–Whitney U test, *p* = 0.002) (Table 4).

### 3.5. Influence of GCS, Serum Creatinine, UCR, and Functional Outcome Measure by FIM

The strongest significant positive correlations were observed between GCS and FIM at admission (Spearman’s ρ = 0.488, *p* < 0.001) and at discharge (ρ = 0.340, *p* < 0.001). Serum creatinine at admission showed a strong positive correlation with admission FIM (ρ = 0.427, *p* < 0.001), as well as moderate positive correlations with discharge FIM (ρ = 0.374, *p* < 0.001) and ΔFIM (ρ = 0.284, *p* < 0.001). A significant negative correlation was found between the UCR at discharge and discharge FIM (ρ = −0.262, *p* < 0.003), as well as with ΔFIM (ρ = −0.207, *p* = 0.02). Higher UCR values were associated with poorer functional outcomes at discharge and less improvement in FIM during inpatient rehabilitation (Table 5). In the subgroup of patients with UCR ≥ 80, FIM at discharge was significantly lower compared with patients with UCR < 80 [median 40 vs. 27; difference −9; 95% CI −20 to 0; Mann–Whitney U test, *p* = 0.02].

### 3.6. Influence of Oral Food Intake on Serum Creatinine Values and UCR and Functional Outcome Measured by FIM

In the subgroup of patients with oral food intake, we found significantly higher serum creatinine values at admission and discharge (Mann–Whitney U test, *p* < 0.001) along with lower UCR values at discharge (Mann–Whitney U test, *p* = 0.007). Patients taking in food orally exhibited significantly higher FIM scores at admission and discharge compared with patients with an NGT/PEG (Mann–Whitney U test, *p* < 0.001 for all comparisons). Concurrently, the duration of rehabilitation was significantly longer in the group of patients with an NGT/PEG (Mann–Whitney U test, *p* < 0.001).

### 3.7. Influence of Admission Serum Creatinine on Functional Independence Measure Through FIM

A hierarchical regression analysis was performed to examine predictors of functional independence at discharge (FIM). Multivariable models were adjusted for age, sex, GCS score, time from injury to inpatient rehabilitation admission, renal function (eGFR), nutritional route (NGT/PEG), level of consciousness, tracheostomy, surgical treatment, multiple subcortical contusions, and FIM at admission. In both models, the FIM value on admission and nutritional route (NGT/PEG) remained significant independent predictors of the FIM at discharge. The level of consciousness was also independently associated with the discharge FIM. After adjustment for clinical and rehabilitation-related covariates, neither the serum creatinine nor UCR on admission retained an independent association with discharge FIM. The addition of UCR to the adjusted model resulted in only a minimal increase in the explained variance (ΔR^2^ = 0.014), indicating a limited incremental prognostic value beyond established clinical predictors. The high overall explained variance was largely driven by the baseline functional status measured by the FIM on admission (Table 6).

## 4. Discussion

The results of this study confirm a correlation of the UCR with FIM in TBI patients during the first 6 months post-injury but do not confirm UCR as an independent factor influencing the FIM at discharge. A study on severe TBI patients that investigated the influence of blood urea nitrogen, creatinine, and other routine blood tests as predictors of outcome also failed to find significant associations with investigated unfavorable outcomes [32]. However, another relevant study found that the blood urea nitrogen-to-albumin ratio may predict mortality in TBI patients [33]. In the literature, the UCR is considered a biomarker of overall protein catabolism. This assumption is based on the fact that elevated plasma urea reflects overall protein breakdown and increases with heightened proteolysis, while low serum creatinine reflects absolute skeletal muscle mass, as it is mainly a product of skeletal muscle breakdown [18]. TBI patients may also develop hypermetabolism, hypercatabolism and malnutrition during rehabilitation [11,12,14]. We used the UCR as a laboratory marker to investigate its potential association with functional outcomes. Our data indicate that a higher UCR at admission is associated with a lower discharge FIM and with less improvement in functional status during inpatient rehabilitation. Patients with a metabolic state leading to higher UCRs at rehabilitation admission will have not only a higher need for assistance with everyday tasks following discharge but also restricted improvement in functional status in the rehabilitation setting during the first 6 months post-injury. A secondary analysis of the Effect of Early Nutritional Support on Frailty, Functional Outcomes, and Recovery of Malnourished Medical Inpatients Trial (EFORT), which included hospitalized patients, showed increased mortality and other adverse events among patients at nutritional risk [20]. Within our cohort, during rehabilitation, there were three fatal outcomes (1%) and the majority of our study population (63%) was classified as having severe TBI. We used FIM as a measure of outcomes. This measure is often used to assess outcomes during inpatient rehabilitation [34], and FIM is a reliable and valid measure of disability in TBI patients, describing not only physical but also global disability due to its cognitive domain [35,36]. Higher FIM scores are important because a single-point increase in the total FIM score can result in about five minutes less assistance needed from another person for basic daily activities [37]. In our study, the FIM an admission remained a significant independent predictor of its value at discharge. Patients with better functional status at rehabilitation commencement had better functional outcomes at discharge and required less help from another person with everyday activities. In this FIM association, patients’ characteristics and clinical and rehabilitation-related covariates did not have additional influence on the result. Within our cohort, we also found a correlation between the serum creatinine at admission and the FIM at both timepoints (admission and discharge); however we did not account for creatine phosphokinase being a muscle marker and its role in creatine phosphate metabolism, which influences serum creatinine levels [38]. When considering the influence of both creatinine and the UCR on functional outcomes, a limitation of our study is that we did not explore the influence of nutritional supplementation, i.e., enteral therapy, on its values. A systematic review and meta-analysis evaluating TBI patients and the effect of nutritional support on clinical outcomes found that those receiving nutritional support showed significant differences in serum protein levels and that nutritional support reduced infection rates (total and lung) and length of stay in the intensive care unit [39]. The influence of nutritional status on functional outcomes has been investigated, but there is still a lack of prospective interventional studies regarding the nutritional management of this population during the rehabilitation period [40]. A notable prospective observational study of patients with TBI showed that those who received enteral nutrition for more than 25% of their rehabilitation stay had higher FIM scores (for both motor and cognitive components) and experienced lower body weight loss [41]. Another important consideration is that due to their injuries, TBI patients have elevated levels of nonspecific inflammation [42,43,44,45]. Additionally, hospitalized patients with higher levels of inflammation benefit less from nutritional therapy as concluded in the previously mentioned EFORT Trial [20]. Conversely, high protein nutrition may sometimes contribute to an elevated UCR [46]. Changes in the UCR as a catabolic marker have been investigated more in patients within the intensive care setting. In the persistent critical illness population, a scoping review and meta-analysis showed that a UCR of approximately 80 mmol/L (equivalent to a BUN-to-creatinine ratio ≥ 20 mg/dL) at intensive care unit admission was associated with a higher risk of in-hospital mortality compared with a value < 80 (equivalent to a BUN-to-creatinine ratio < 20 mg/dL) [22]. At our hospital, TBI patients are most often transferred from intensive care units and post-intensive neurosurgical care units to inpatient rehabilitation; they may also develop malnutrition during their stay in intensive care [47]. An important finding of this study was the poorer outcomes for patients with a UCR ≥ 80; these patients had significantly lower FIM scores at discharge compared to those with a UCR < 80. This cut-off was predetermined based on research conducted in patients with persistent critical illness; therefore, in the TBI setting, it should be interpreted as exploratory. Although we found a correlation between the UCR and FIM, and specifically at UCR values ≥ 80, after adjustment for patient characteristics and clinical and rehabilitation-related covariates, the UCR was no longer independently associated with the FIM at discharge. The high overall explained variance was largely driven by the baseline functional status measured as the FIM at admission. Nevertheless, the clinical relevance of a UCR ≥ 80 still remains. Individuals with this high a UCR (≥80) will have worse functional outcomes and will need a higher level of assistance in everyday living. Regarding the nutritional route, we also considered a subgroup of patients, with and without oral intake. Patients without a feeding tube had significantly higher FIM scores at admission and discharge than those with a feeding tube (NGT or PEG). This cohort also had significantly higher serum creatinine levels at both assessments and a lower UCR at admission. However, patients with a feeding tube (NGT or PEG) had longer hospital stays and a significantly higher UCR at discharge compared to patients without a feeding tube. The route of nutritional therapy (oral, NGT, PEG, parenteral nutrition) in TBI patients is an important factor to address [48]. In our study, we found that the nutritional route, i.e., NGT/PEG, is a significant independent predictor of the discharge FIM. This means that the previously mentioned association remained significant after adjusting for patient characteristics and clinical and rehabilitation-related covariates. From a clinical perspective, patients with feeding tubes represent a subset of severely injured individuals with severe neurological deficits, including dysphagia. It is known that patients with dysphagia can develop many complications, for example, they are at risk of developing pulmonary aspiration, as an acute complication, or aspiration pneumonia, excessive secretion, and weight loss, as chronic complications [49]. Patients with feeding tubes (NGT or PEG), i.e., patients with dysphagia display a metabolic state that causes higher UCRs at discharge compared to those without feeding tubes. All of the above-mentioned factors are not only clinical considerations for the patient; they also provide clinicians with additional prognostic information regarding outcomes. The results of our study indicate another important predictor of outcomes, the level of consciousness. Patients with DoC have a much lower FIM value, both at admission and at discharge, in comparison with those who have normal levels of consciousness. We can interpret that patients with DoC will have worse functional outcomes and will require more assistance in everyday living. Nevertheless, clinicians need to be cautious when explaining a patient’s prognosis to their family members. Populations of patients with DoC also exhibit expected functional improvements, and up to 40% of this population can reach partial or full independence [50,51]. Overall, TBI patients represent a complex patient population. The pathophysiology of brain damage involves tissue injury at the molecular, cellular, and signalling levels, thereby affecting various aspects of brain physiology [42,43] and can extend to systemic organ dysfunction. Furthermore, the initial GCS score, which describes the neurological status at the onset of injury, has a strong significant positive correlation with the FIM at both timepoints (admission and discharge). Once more, the correlation between the GCS and the FIM highlights the critical impact of early clinical status and aligns with previous studies that have confirmed the prognostic value and validity of the GCS in predicting functional outcomes in TBI population [52,53,54,55,56]. Although our study included only TBI patients, this research does not clarify whether the relationship between UCR and FIM reflects mechanisms specific to this patient population or whether it is a general manifestation of systemic catabolic burden in severely injured patients. Gastrointestinal, metabolic and endocrine dysregulations can develop following stroke [57,58,59] and other neurological diseases [60], and may influence patients outcomes [61,62,63]. Therefore, a limitation of our study is the lack of comparison with other neurological disorders.

## 5. Conclusions

The UCR influences functional outcomes measured using the FIM, in TBI patients during the first 6 months post-injury; however, we cannot consider the UCR as an independent predictor of functional outcomes. Rather, the FIM at admission, nutritional route (NGT/PEG), and level of consciousness remained the independent predictors of functional outcome. Nevertheless, during inpatient rehabilitation, nutritional status—encompassing laboratory parameters—should be closely monitored, and the UCR could be considered a marker of global catabolic burden. More studies are needed to investigate further potential associations between specific laboratory values and the overall nutritional status, as well as functional outcomes.

## 6. Limitations

This study has several limitations. First, the relatively high explained variance may largely reflects the strong contribution of the baseline FIM, which may limit the incremental influence of other predictors. After adjustment for clinical and rehabilitation-related covariates, the serum creatinine and UCR were no longer independently associated with FIM at discharge. Furthermore, inflammatory biomarkers and objective muscle mass indicators were not available and therefore could not be included in the multivariable models. Missing data were handled using a complete-case approach, and no imputation procedures were performed. Finally, the observational design of this study precludes causal inference, and residual confounding cannot be entirely ruled out.

## Figures and Tables

**Table 1:** Baseline demographic, clinical, neuroradiological, and functional characteristics of the study population.

Patients Characteristics	
Gender, n 291 (%)	
Male	239 (82.1)
Female	52 (17.9)
Age (years), median (IQR)	45 (27–57)
Time from injury to rehabilitation admission (days), median (IQR)	66 (49–97)
Length of inpatient rehabilitation (days), median (IQR)	82 (42–175)
Treatment, n 287 (%)	
Conservative	145 (50.5)
Neurosurgical operation	142 (49.5)
Level of consciousness at admission, n 291 (%)	
Conscious	235 (80.8)
Impaired consciousness	56 (19.2)
Feeding method, n 291 (%)	
Oral feeding	211 (72.5)
Nasogastric tube (NGT)	56 (19.2)
Percutaneous endoscopic gastrostomy (PEG)	24 (8.2)
Tracheostomy, n 291 (%)	
Yes	63 (21.6)
No	114 (39.2)
Removed	114 (39.2)
Glasgow Coma Scale (GCS), median (IQR)	7 (4–11)
Severity of brain injury (n = 285), n (%)	
Mild: loss of consciousness or PTA < 30 min or GCS 13–15	59 (21.0)
Moderate: loss of consciousness or PTA 30 min–24 h or GCS 9–12	45 (16.0)
Severe: loss of consciousness or PTA > 24 h or GCS 3–8	177 (63.0)
Functional Independence Measure (FIM), median (IQR)	37 (20–95)
CT findings, n (%)	
Contusion	219 (75.8)
Subarachnoid hemorrhage (SAH)	199 (68.9)
Subdural hematoma (SDH)	162 (56.1)

FIM—functional independence measure; GCS—Glasgow Coma Scale; PTA—post-traumatic amnesia; NGT—nasogastric tube; PEG—percutaneous endoscopic gastrostomy; IQR—interquartile range. Categorical data are described with n (%) and continuous data with median and IQR.

**Table 2:** Changes in laboratory parameters during rehabilitation.

	n	Median (IQR)	^†^ Difference	95% CI	*p* * Value
Serum urea					
Admission	122	3.2 (2.4–4.6)	0.6	0.3 to 0.9	**0.001**
Discharge	122	4.1 (3.2–4.9)			
Serum creatinine					
Admission	137	57 (47–71)	5	2.5 to 8	**<0.001**
Discharge	137	64 (54–73)			
Urea-to-creatinine ratio (UCR)					
Admission	122	61.0 (45.5–72.4)	4.6	−0.7 to 10.2	0.09
Discharge	122	63.2 (51.1–80.9)			

UCR—urea-to-creatinine ratio; CI—confidence interval; IQR—interquartile range; * Wilcoxon test; ^†^ Hodges–Lehmann estimator of the median difference. Different n values reflect unavailable paired measurements for some patients. **Bold** values indicate statistical significance.

**Table 3:** Changes in functional independence during rehabilitation.

Functional Independence Measure (FIM)	n	Median (IQR)	^†^ Difference	95% CI	*p* * Value
All patients					
Admission	291	37 (20–95)	21	17 to 26	**<0.001**
Discharge	291	93 (36–112)			
Conscious patients					
Admission	235	47 (26–102)	25	20 to 30	**<0.001**
Discharge	235	102 (63–114)			
Patients with impaired consciousness					
Admission	56	18 (18–18) (18–48)	7	3 to 13	**<0.001**
Discharge	56	19 (18–36) (18–106)			

FIM—functional independence measure; CI—confidence interval; IQR—interquartile range; * Wilcoxon test; ^†^ Hodges–Lehmann estimator of the median difference. **Bold** values indicate statistical significance.

**Table 4:** Clinical and laboratory characteristics according to treatment modality.

	Median (IQR)		^†^ Difference	95% CI	*p* *
	Conservative Treatment	Neurosurgical Operation			
Glasgow Coma Scale (GCS)	6 (4–10)	8 (4–12)	1	0 to 2	0.15
FIM at admission	39 (21.5–98)	33 (19–88)	−2	−7 to 0	0.14
FIM at discharge	101 (47–115.5)	82.5 (30.8–110)	−9	−16 to −3	**0.002**
Serum urea	3.7 (2.6–4.7)	3.7 (2.6–4.8)	0.1	−0.3 to 0.4	0.73
Serum creatinine	62 (53–73)	63 (51–74.5)	0	−5 to 4	0.87
UCR	58.9 (42.8–70.5)	56 (43.9–71)	0.31	−5.2 to 5.3	0.91
Duration of inpatient rehabilitation	91 (42–181)	75 (35–160)	−11	−28 to 4	0.17

IQR—interquartile range; UCR—urea-to-creatinine ratio; FIM—functional independence measure; CI—confidence interval; * Mann–Whitney U test; ^†^ Hodges–Lehmann estimator of the median difference. **Bold** values indicate statistical significance.

**Table 5:** Correlations of clinical and laboratory variables with functional independence outcomes.

	Spearman’s ρ (*p* Value)		
	Admission FIM	Discharge FIM	ΔFIM
Serum urea at admission	**0.198 (0.002)**	0.051 (0.43)	−0.052 (0.42)
Serum creatinine at admission	**0.427 (<0.001)**	**0.353 (<0.001)**	0.030 (0.63)
UCR admission	−0.054 (0.38)	**−0.155 (0.01)**	−0.114 (0.07)
Glasgow Coma Scale (GCS)	**0.488 (<0.001)**	**0.340 (<0.001)**	−0.094 (0.12)
Serum urea at discharge	-	0.058 (0.52)	0.067 (0.46)
Serum creatinine at discharge	-	**0.374 (<0.001)**	**0.284 (0.001)**
UCR at discharge	-	**−0.262 (0.003)**	**−0.207 (0.02)**

UCR—urea-to-creatinine ratio; FIM—functional independence measure. ΔFIM—difference between discharge and admission FIM scores. **Bold** values indicate statistical significance.

**Table 6:** Creatinine and UCR on admission as predictors of functional independence at discharge.

	Model I (Clinical Covariates)			Model II (Clinical Covariates + Creatinine)		
	β	95% CI for ß	*p* Value	β	95% CI for ß	*p* Value
Age	−0.58	−0.9 to −0.3	**<0.001**	−0.43	−1.1 to 0.3	0.24
Gender	6.41	−1.3 to 14.2	0.10	8.86	−4 to 21.7	0.18
Glasgow Coma Scale (GCS)	0.22	−0.7 to 1.1	0.64	0.22	−0.7 to 1.1	0.63
Time from injury to IR	0.00	0 to 0	0.81	0.00	0 to 0	0.79
eGFR	−0.07	−0.4 to 0.3	0.69	0.15	−0.8 to 1.1	0.77
NGT/PEG	−27.52	−37.7 to −17.3	**<0.001**	−27.40	−37.6 to −17.2	**<0.001**
Level of consciousness	−16.53	−26.2 to −6.9	**0.001**	−16.50	−26.1 to −6.8	**0.001**
Tracheostomy	3.98	−2.8 to 10.8	0.25	3.72	−3.2 to 10.6	0.29
Surgical treatment	−1.16	−6.9 to 4.6	0.69	−1.18	−7 to 4.6	0.69
Multiple subcortical contusions	−0.71	−7.3 to 5.8	0.83	−0.77	−7.3 to 5.8	0.82
FIM at admission	0.57	0.5 to 0.7	**<0.001**	0.57	0.5 to 0.7	**<0.001**
Creatinine at admission	-	-	-	0.17	−0.6 to 0.9	0.64
Regression model (dependent variable: FIM at discharge)	R = 0.844; R^2^ = 0.712; R^2^_adj_ = 0.697; F_(11, 212)_ = 47.6; *p* < 0.001			R = 0.844; R^2^ = 0.712; R^2^_adj_ = 0.696; F_(12, 211)_ = 43.5; *p* < 0.001		
	ΔR^2^ = 0.00; Cohen’s f^2^ = 0.00					
	**Model I** **(clinical covariates)**			**Model II** **(clinical covariates + UCR)**		
Age	−0.58	−0.9 to −0.3	**<0.001**	−0.54	−0.9 to −0.2	**0.001**
Gender	6.41	−1.3 to 14.2	0.10	6.86	−0.9 to 14.6	0.08
Glasgow Coma Scale (GCS)	0.22	−0.7 to 1.1	0.64	0.27	−0.6 to 1.2	0.56
Time from injury to IR	0.00	0 to 0	0.81	0.00	0 to 0	0.91
eGFR	−0.07	−0.4 to 0.3	0.69	−0.02	−0.4 to 0.3	0.92
NGT/PEG	−27.52	−37.7 to −17.3	**<0.001**	−27.07	−37.3 to −16.9	**<0.001**
Level of consciousness	−16.53	−26.2 to −6.9	**0.001**	−16.45	−26 to −6.9	**0.001**
Tracheostomy	3.98	−2.8 to 10.8	0.25	3.57	−3.2 to 10.4	0.30
Surgical treatment	−1.16	−6.9 to 4.6	0.69	−0.65	−6.4 to 5.1	0.83
Multiple subcortical contusions	−0.71	−7.3 to 5.8	0.83	−0.83	−7.4 to 5.7	0.80
FIM at admission	0.57	0.5 to 0.7	**<0.001**	0.57	0.5 to 0.7	**<0.001**
Urea-to-creatinine ratio (UCR) at admission	-	-	-	−0.10	−0.2 to 0	0.08
Regression model (dependent variable: FIM at discharge)	R = 0.844; R^2^ = 0.713; R^2^_adj_ = 0.697; F_(11, 210)_ = 47.6; *p* < 0.001			R = 0.847; R^2^ = 0.717; R^2^_adj_ = 0.700; F_(12, 209)_ = 44.0; *p* < 0.001		
	ΔR^2^ = 0.004; Cohen’s f^2^ = 0.014					

CI—confidence interval. R^2^_adj_—adjusted coefficient of determination; f^2^. Cohen’s effect size (0.02 small; 0.15 medium; 0.35 large). **Bold** values indicate statistical significance.

## Data Availability

The data supporting the findings of this study are available from the corresponding author upon reasonable request. The data are not publicly available due to their use in another ongoing analyses.
